# Rewiring Pyroptosis to Potentiate Cancer Immunotherapy via a Gasdermin D Agonist Bypassing Caspase‐3

**DOI:** 10.1002/advs.76655

**Published:** 2026-07-23

**Authors:** Dan Zhao, Lingling Zhang, Yue Wen, Lianghui Cheng, Sidan Tian, Fanling Meng, Liang Luo

**Affiliations:** ^1^ National Engineering Research Center for Nanomedicine College of Life Science and Technology and Key Laboratory of Molecular Biophysics of the Ministry of Education Huazhong University of Science and Technology Wuhan China; ^2^ Department of Gastroenterology Tongji Hospital of Tongji Medical College Huazhong University of Science and Technology Wuhan China; ^3^ Hubei Key Laboratory of Bioinorganic Chemistry and Materia Medica School of Chemistry and Chemical Engineering Huazhong University of Science and Technology Wuhan China; ^4^ Hubei Key Laboratory of Bioinformatics and Molecular Imaging College of Life Science and Technology and Key Laboratory of Molecular Biophysics of the Ministry of Education Huazhong University of Science and Technology Wuhan China

**Keywords:** bypassing Caspase3, glutathione deplesion, immunotherapy, oxidative stress, pyroptosis

## Abstract

Pyroptosis holds great promise for evoking robust anti‐tumor immunity, but many pyroptosis induction strategies rely on caspase‐3 (Casp‐3) activation and face challenges of the immunosuppressive nature of apoptosis and the frequent silencing of gasdermin E (GSDME), a substrate of Casp‐3, in tumors. Here, we report a strategy to rewire the pyroptotic pathway by bypassing Casp‐3 to directly activate gasdermin D (GSDMD). We identify a small‐molecule agonist, (E)‐2,3‐diiodobut‐2‐ene‐1,4‐diol (DIBDO), which undergoes deiodination to release iodide ions upon activation by the tumor‐abundant nucleophile glutathione, thereby catalytically generating singlet oxygen and molecular iodine. This cascade induces oxidative damage that downregulates key mediators of caspase‐9 and caspase‐8 pathways, thereby down‐regulating Casp‐3 activation. This mechanism selectively triggers GSDMD‐mediated pyroptosis, provoking robust immunogenic cell death and inflammatory cytokine release to reconfigure the tumor immune microenvironment. In murine tumor models, DIBDO enhances tumor infiltration of cytotoxic T cells and synergizes with checkpoint blockade therapy to suppress both primary and distal tumors. It can also serve as an in situ or exogenous vaccine to elicit potent and durable antitumor immunity. This work presents a paradigm‐shifting approach to cancer immunotherapy by decoupling pyroptosis from Casp‐3 dependence, offering a promising avenue to expand the scope of immunogenic cell death‐based treatments.

## Graphical abstract

This work introduces a small‐molecule agonist, DIBDO, that bypasses Casp‐3 to directly trigger GSDMD‐mediated pyroptosis in tumors. Activated by glutathione, DIBDO generates singlet oxygen and molecular iodine, blocking Casp‐3 pathways and inducing immunogenic cell death. This reconfigures the tumor microenvironment, transforms the tumor cells into in situ or exogenous vaccine, offering a novel paradigm for cancer immunotherapy.

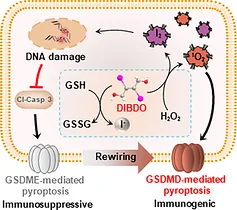

## 1 Introduction

Tumor immunotherapy, which harnesses the immune system to recognize and eliminate malignant cells, has emerged as a breakthrough in oncotherapy and garnered significant attention, yet its clinical application confronts persistent challenges, including inadequate immune responses and systemic toxicity from nonspecific immune activation [1, 2, 3, 4]. Pyroptosis, a specialized form of programmed cell death defined by cellular membrane disruption and intense inflammatory reactions [5, 6, 7], has been identified as a pivotal target in cancer immunotherapy [8], owing to its essential function in reconfiguring the tumor immune microenvironment [9, 10, 11, 12]. Pyroptosis is orchestrated by the gasdermin (GSDM) family of pore‐forming proteins [13, 14, 15, 16, 17, 18, 19, 20]. The cleavage of GSDM proteins, particularly GSDMD and GSDME, is essential for initiating pyroptosis and boosting antitumor immunity [13, 19, 21, 22]. However, GSDME is usually silenced or mutated in cancers [19, 23, 24, 25], and more critically, it is identified as a substrate of caspase‐3 (Casp‐3), a primary executioner protease in apoptosis [26, 27, 28, 29]. The activation of Casp‐3 can trigger this immunologically quiescent cell death, and may even foster tolerance to tumor antigens.

Conversely, GSDMD, another key executioner protein in pyroptosis [17, 20, 30], is frequently observed to be expressed in tumors [23, 31, 32]. Moreover, small molecules such as simvastatin can induce GSDMD activation in tumor types with low basal expression of inflammasome components (such as caspase‐1 [33, 34]), supporting the pan‐tumor therapeutic potential of targeting the GSDMD axis. This implies that targeting GSDMD‐mediated pyroptosis represents a potential therapeutic avenue to bypass the limitations inherent in Casp‐3/GSDME‐dependent pathways. Such a paradigm shift may enable more precise induction of immunogenic cell death while minimizing collateral toxicity, thereby effectively overcoming the immunosuppressive tumor microenvironment (TME) characteristic of cold tumors. On the other hand, emerging evidence has revealed that molecular iodine (I_2_) can damage intracellular proteins and DNA [35, 36], inducing cell death through a process independent of Casp‐3/‐8/‐9 [33, 34]. In addition, the rapid and extensive oxidation of glutathione (GSH), a critical reducing agent overexpressed in many tumor cells [37, 38, 39], to glutathione disulfide (GSSG) could also inhibit cytochrome c (Cyt C)‐mediated caspase‐9 (Casp‐9) and Casp‐3 activation [40]. Collectively, these findings highlight the potential of circumventing the reliance on specific caspases in modulating cell death pathways.

Following these rationales, we identify a GSH‐activatable small molecule, (*E*)‐2,3‐diiodobut‐2‐ene‐1,4‐diol (DIBDO) (Scheme 1), which is water‐soluble, electrically neutral, and physiologically inert. We reveal that DIBDO can rapidly detach iodide ions (I^−^) upon reaction with nucleophilic GSH in the TME. The liberated I^−^ is subsequently converted to I_2_ while catalyzing the decomposition of H_2_O_2_ to generate reactive oxygen species (ROS). Importantly, as a small and neutral molecule with good water solubility, DIBDO can conduct these reactions in proximity to genomic DNA. As a consequence, DIBDO treatment can induce caspase‐1 (Casp‐1) cleavage and cause DNA damage simultaneously, which in turn downregulates the transcription of genes associated with Casp‐3 activation. Together, the resulting pyroptosis is GSDMD‐oriented, and can induce pro‐inflammatory cytokines secretion, evoke robust immunogenic cell death effects, and reverse the immunosuppressive TME, facilitating the transition of immunologically “cold” tumors to “hot” for stimulated cancer immunotherapy.

**SCHEME 1:**
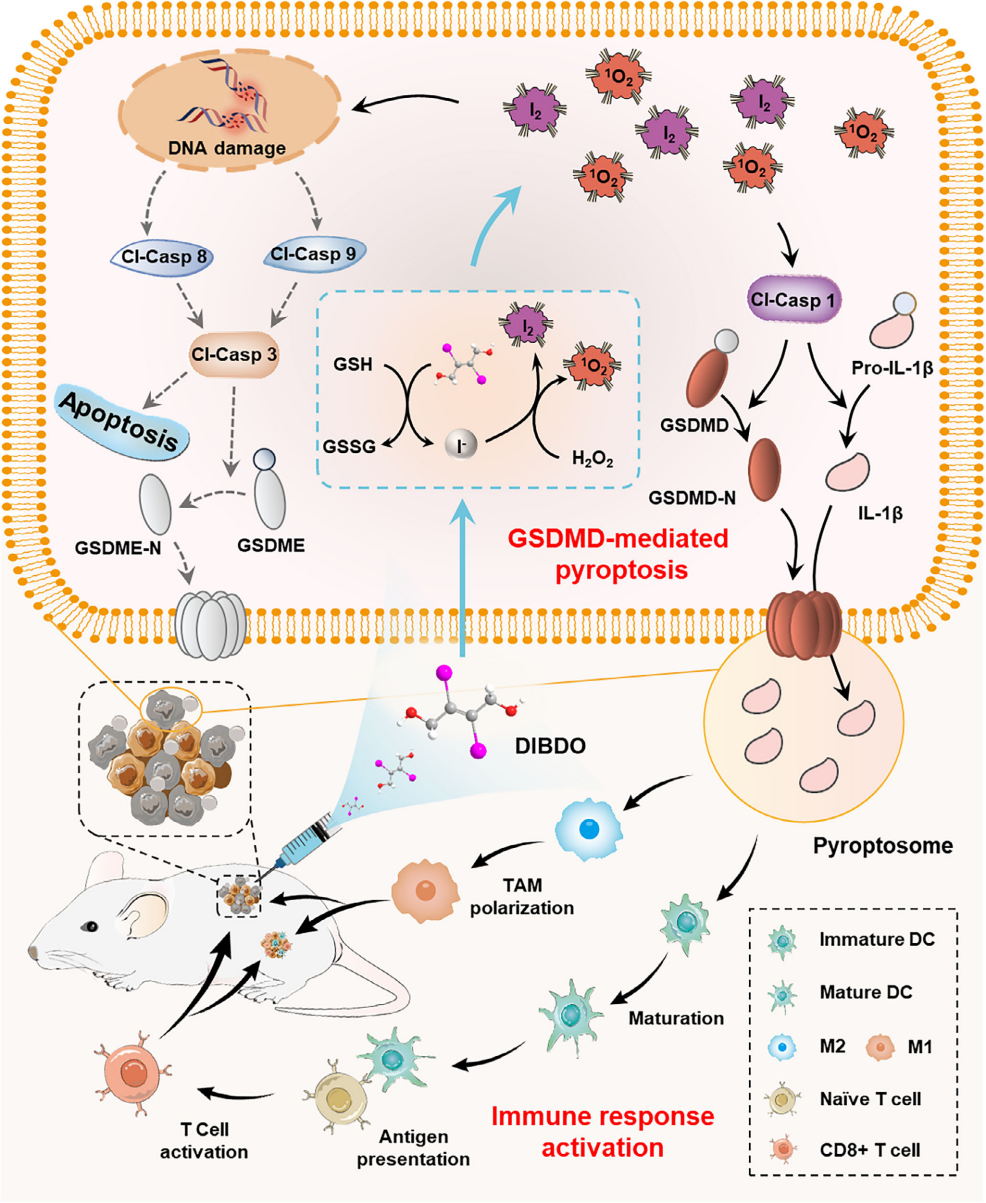
Schematic illustration of DIBDO‐mediated pyroptosis for stimulated antitumor immunotherapy by bypassing Casp‐3 activation.

## 2 Results

### 2.1 DIBDO‐Mediated ROS Generation in TME

As a transdiiodoalkene, the C─I bonds of DIBDO can be cleaved in response to the attack of nucleophiles [41, 42], so that the nucleophilic GSH in TME can trigger the release of iodide ions from DIBDO. As shown in the ^1^H NMR spectra of a mixture of DIBDO and GSH (Figure 1a), after the reaction, the characteristic peak of DIBDO at δ 4.51 ppm disappeared completely, concomitant with the emergence of a new signal at δ 4.29 ppm, which corresponds to but‐2‐yne‐1,4‐diol (DBDO). This process also enabled the conversion of GSH to glutathione disulfide (GSSG), as confirmed by the appearance of a cluster of new peaks at around δ 3.30 ppm. Further, 5,5’‐dithiobis‐(2‐nitrobenzoic acid) (DTNB) was employed as a GSH indicator to track the consumption of GSH, which turned yellow quickly in the presence of GSH (Figure S1). Quantitative monitoring in Figure 1b demonstrates time‐dependent GSH consumption during co‐incubation with DIBDO, culminating in complete depletion after 48 h (Figure S1).

**FIGURE 1:**
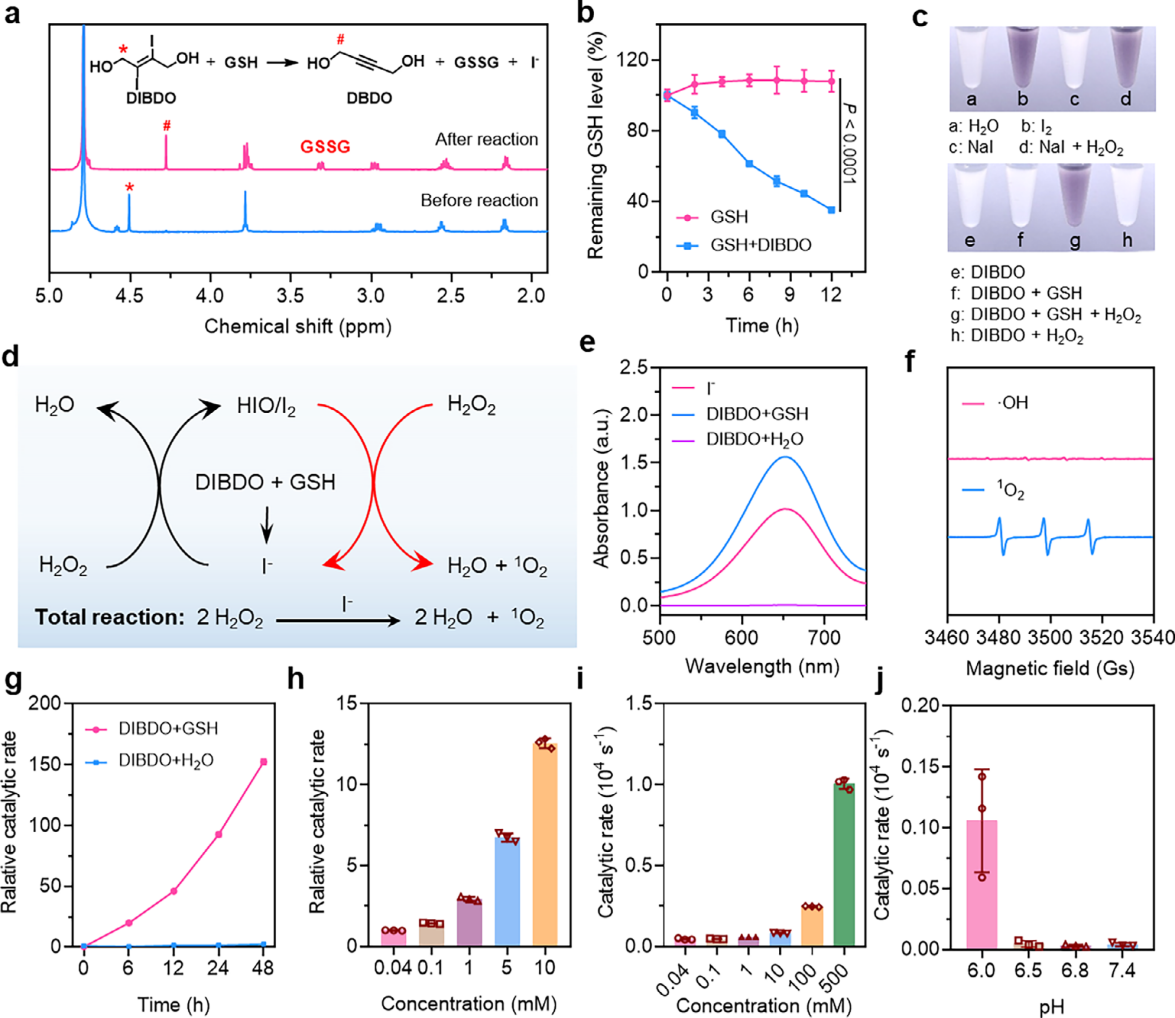
GSH activated deiodination and catalytic performance of DIBDO. (a) The ^1^H NMR spectra of a mixture of DIBDO (1 mM) and GSH (2 mM) in D_2_O before and after reaction. (b) Remaining GSH levels after incubated with or without DIBDO (2 mM) for different times (n = 3, t‐test). (c) Photographs of 1 mL starch solution mixed with H_2_O, I_2_ (20 µL, 5 mM), NaI (50 µL, 4 mM), NaI (50 µL, 4 mM) with H_2_O_2_ (NaI + H_2_O_2_), DIBDO (50 µL, 2 mM), DIBDO (50 µL, 2 mM) with GSH (DIBDO + GSH), DIBDO (50 µL, 2 mM) with GSH and H_2_O_2_ (DIBDO + GSH + H_2_O_2_), and DIBDO (50 µL, 2 mM) with H_2_O_2_ (DIBDO + H_2_O_2_). GSH concentration:10 mM; H_2_O_2_ concentration: 500 mM. (d) Mechanism of the DIBDO/GSH system in catalyzing the conversion of H_2_O_2_ into ^1^O_2_. (e) Absorption spectra of TMB in the presence of NaI + H_2_O_2_, DIBDO + H_2_O_2_, and DIBDO + GSH + H_2_O_2_. NaI concentration: 200 µM; H_2_O_2_ concentration: 500 mM; DIBDO final concentration: 100 µM. (f) EPR spectra of DIBDO + GSH by using DMPO (100 mM, upper) and TEMP (100 mM, below) as a probe of •OH and ^1^O_2_, respectively. (g) Catalytic activity of DIBDO (2 mM) treated with or without GSH (10 mM) for different durations (n = 3). The test conditions: TMB: 50 µg mL^−1^; DIBDO: 50 µM; H_2_O_2_: 500 mM; buffer: NaAc/HAc: 0.2 M, pH 3.6. (h) Catalytic rate of DIBDO (2 mM) incubated with GSH solution of different concentrations for 4 h (n = 3). Test conditions were consistent with those of (g). (i, j) Catalytic rate of DIBDO/GSH system under different H_2_O_2_ concentrations (n = 3) (i) and pH values (n = 3) (j). Final concentration: TMB: 50 µg mL^−1^; DIBDO: 50 µM; NaAc/HAc: 0.2 M. Data = mean ± SD. n = the number of dots as independent replicates in each graph. Statistical analysis was performed using t‐test.

In the presence of H_2_O_2_, which is also highly expressed within the tumor microenvironment, the detached I^−^ from DIBDO can continue to transform into I_2_ while catalyzing the conversion of H_2_O_2_ to ROS. The formation of I_2_ was verified by the classical starch test (Figure S2). As depicted in Figure 1c and Figure S3, the starch solutions developed a blue‐violet color when exposed to I_2_‐generation systems, i.e., NaI + H_2_O_2_ and DIBDO + GSH + H_2_O_2_. In contrast, no color change occurred with NaI or DIBDO alone. Consistently, the introduction of DIBDO into AgNO_3_ solution yielded no precipitate, whereas the coincubation products of DIBDO and GSH produced abundant yellow precipitates (Figure S4). Collectively, these results established that GSH induced sustained I^−^ release from DIBDO, followed by rapid I_2_ generation upon H_2_O_2_ exposure.

We next examined the activity of the DIBDO/GSH system in catalyzing the conversion of H_2_O_2_ into ROS, singlet oxygen (^1^O_2_) in the majority (Figure 1d) [43]. Two ROS probes, 3,3’,5,5’‐tetramethylbenzidine (TMB) and 2’,7’‐dichlorodihydrofluorescein (DCF), were rapidly oxidized when H_2_O_2_ was co‐incubated with the mixed solution of DIBDO and GSH (Figure 1e and Figure S5), as demonstrated by the characteristic absorbance at 652 nm and fluorescence emission at 525 nm, respectively. As a comparison, DIBDO itself exhibited no catalytic activity under identical conditions. Subsequent kinetic analysis revealed a direct correlation between substrate consumption rate and DIBDO concentration within the catalytic mixture (Figure S6). Notably, the catalytic activity of the DIBDO/GSH system did not show significant change across varied buffer conditions (Figure S7), indicating the stability in various physiological conditions. Of note, electron paramagnetic resonance (EPR) spectroscopy detected signals exclusively attributable to ^1^O_2_, with no evidence of hydroxyl radical (•OH) generation (Figure 1f). These observations were corroborated using terephthalic acid (TA) and 9,10‐anthracenediyl‐bis(methylene)dimalonic acid (ABDA) as specific probes for •OH and ^1^O_2_ detection, respectively (Figure S8).

Since both DIBDO and its deiodination product DBDO did not catalyze H_2_O_2_ into ROS, and the catalytic rate of I^−^ exhibited a linear concentration dependence (Figure S9), we evaluated iodine release from DIBDO based on time‐dependent I^−^ catalytic kinetics. Iohexol, a non‐deiodinable compound, served as the control (Figure S10). As shown in Figure 1g, extending the incubation time of DIBDO and GSH to 48 h enhanced the catalytic rate for over 150 folds, while increasing GSH concentration from 0.04 mM to 10 mM during 4 h incubation yielded a 12‐fold catalytic efficiency increase (Figure 1h). These results collectively demonstrated the sustained iodide release from DIBDO. The ROS generation catalyzed by the DIBDO/GSH system could also be promoted under increased H_2_O_2_ concentrations and reduced pH values (Figure 1i,j). These findings suggested that the requirements to activate and amplify DIBDO's catalytic performance precisely aligned with the characteristics of TME, i.e., elevated GSH/H_2_O_2_ levels and weak acidity, which strongly indicated DIBDO's potential for tumor‐specific therapy.

### 2.2 In Vitro Antitumor Efficacy of DIBDO

To assess the in vitro antitumor performance of DIBDO, we measured the catalytic activity in lysates from DIBDO‐treated HeLa cells using TMB as the indicator, with iohexol and NaI serving as controls (Figure S11). Lysates of DIBDO‐treated HeLa cells exhibited significantly higher catalytic activity than those treated with NaI or iohexol. This result confirmed the structural advantage of DIBDO, as iohexol lacked deiodination capability and transmembrane transport of I^−^ into living cells occurred primarily through specific symporters under strict cellular regulation [44]. Subsequent quantification of residual intracellular GSH and H_2_O_2_ concentrations following a 4 h DIBDO incubation showed that the GSH/GSSG ratio decreased significantly with increasing concentrations of DIBDO (Figure 2a), indicating rapid GSH depletion and the potential induction of oxidative stress. Concurrent H_2_O_2_ consumption was observed (Figure 2b), consistent with GSH‐mediated iodide release from DIBDO and subsequent iodine‐catalyzed H_2_O_2_ decomposition in vitro.

**FIGURE 2:**
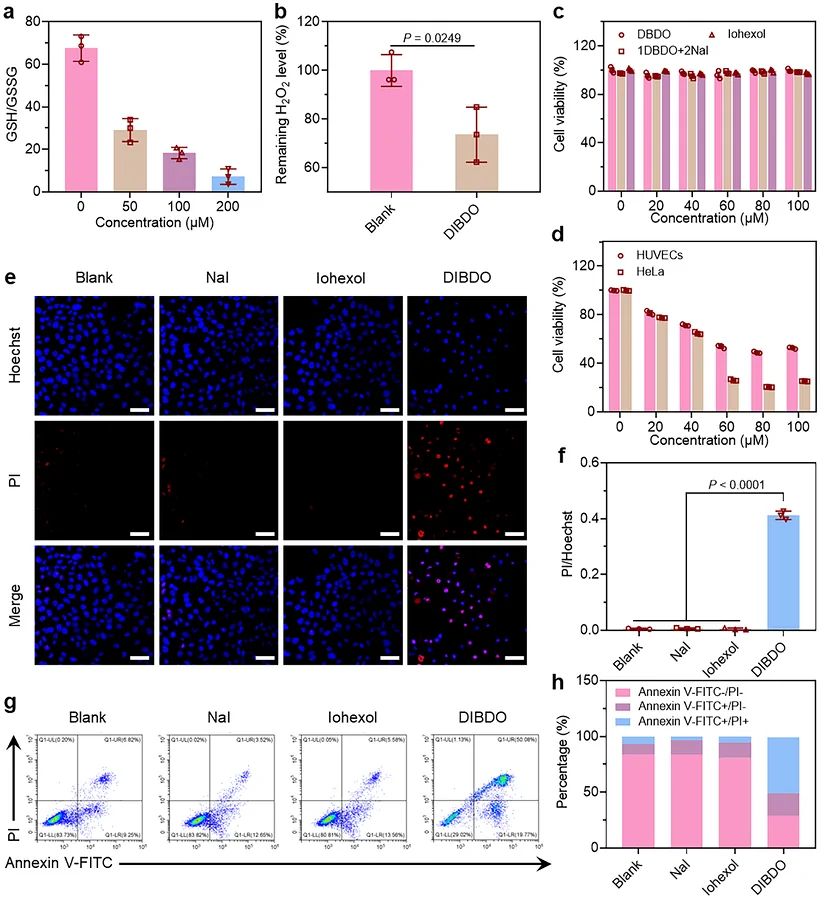
In vitro antitumor activity of DIBDO. (a) The GSH/GSSG ratio in HeLa cells after 4 h of treatment with DIBDO (200 µM) at various concentration (*n* = 3). (b) Remaining H_2_O_2_ level in HeLa cells after 4 h of treatment with DIBDO (200 µM) (*n* = 3, t‐test). (c) Cell viability of HeLa cells treated with DBDO, iohexol, and a mixture of DBDO with twice the equivalent of NaI at various concentrations (*n* = 3). (d) Cell viability of HeLa cells and HUVECs treated with DIBDO at different concentrations (*n* = 3). (e, f) PI staining (e) and quantitative analysis (f) of HeLa cells after treatment with NaI (400 µM), iohexol (200 µM), and DIBDO (200 µM) (*n* = 3, ANOVA) for 12 h. Scale bar: 50 µm. (g, h) Flow cytometry analysis (g) and corresponding quantitative analysis (h) of HeLa cells receiving various treatment (400 µM of NaI, 200 µM of iohexol, and 200 µM of DIBDO, respectively) and co‐stained with Annexin V‐FITC and PI. Data = mean ± SD. n = the number of dots as independent replicates in each graph. Statistical analysis was performed using one‐way ANOVA or t‐test.

We next investigated the antitumor performance of DIBDO. The degradation products of DIBDO in the presence of GSH, i.e., DBDO and NaI, showed negligible cytotoxicity toward cells (> 90% cell viability at 100 µM, Figure 2c), aligning with their minimal catalytic activity. Conversely, DIBDO caused over 70% viability reduction of HeLa cells at 100 µM, demonstrating significant cytotoxicity (Figure 2d). Notably, more than 50% of human umbilical vein endothelial cells (HUVECs) remained viable under identical DIBDO treatment, indicating DIBDO exhibited differential toxicity toward tumor cells and normal cells. In hoechst/propidium iodide (PI) co‐staining assays, DIBDO‐treated HeLa cells displayed markedly enhanced red fluorescence signals compared to NaI‐ or iohexol‐treated cells (Figure 2e,f), signifying potent induction of membrane integrity loss and late‐stage cell death. Flow cytometry analysis (Annexin V‐FITC/PI staining) confirmed that over 50% of DIBDO‐treated HeLa cells were in late‐stage death, versus less than 7% in control groups (Figure 2g,h). Collectively, these findings provide a mechanistic basis for DIBDO‐mediated, GSH‐activated antitumor efficacy.

### 2.3 DIBDO Blocks Casp‐3 Activation and Induces GSDMD‐Mediated Pyroptosis in Tumor Cells

We first utilized 2’,7’‐dichlorodihydrofluorescein diacetate (DCFH‐DA) as a probe to assess intracellular oxidative stress change. As anticipated, HeLa cells treated with DIBDO exhibited green fluorescence signals twice as intense as those exposed to NaI or iohexol (Figure 3a and Figure S12), confirming significantly elevated oxidative stress relative to controls. Furthermore, excessive ROS and I_2_ can damage intracellular proteins, DNA, and other cellular components, impairing critical organelle functions and disrupting normal physiological metabolic processes [35, 45, 46, 47]. Therefore, we quantified protein concentrations and DNA content in DIBDO‐treated HeLa cells. The results demonstrated significant decreases in both parameters after short‐term DIBDO exposure (Figure 3b and Figure S13), indicating the damage of proteins and DNA. Additionally, Fluo 4‐AM labeling assay revealed an over 50‐fold increase in cytosolic Ca^2^^+^ fluorescence intensity in DIBDO‐treated cells versus untreated controls (Figure 3c and Figure S14). This suggested that endoplasmic reticulum damage occurred and triggered massive Ca^2+^ release within cells [48, 49], which propagated mitochondrial dysfunction. Consequently, we employed 5,5’,6,6’‐tetrachloro‐1,1’,3,3‐tetraethylbenzimidazolocarbocyanine iodide (JC‐1) to evaluate mitochondrial membrane potential, the dissipation of which immediately followed mitochondrial structural/functional compromise. In NaI‐ and iohexol‐treated cells, JC‐1 accumulated in mitochondria as J‐aggregates and emitted red fluorescence [50, 51, 52, 53], reflecting preserved mitochondrial membrane potential (Figure 3d). Conversely, DIBDO treatment converted JC‐1 to green‐fluorescent monomers, indicating mitochondrial depolarization driven by synergistic ROS and calcium overload.

**FIGURE 3:**
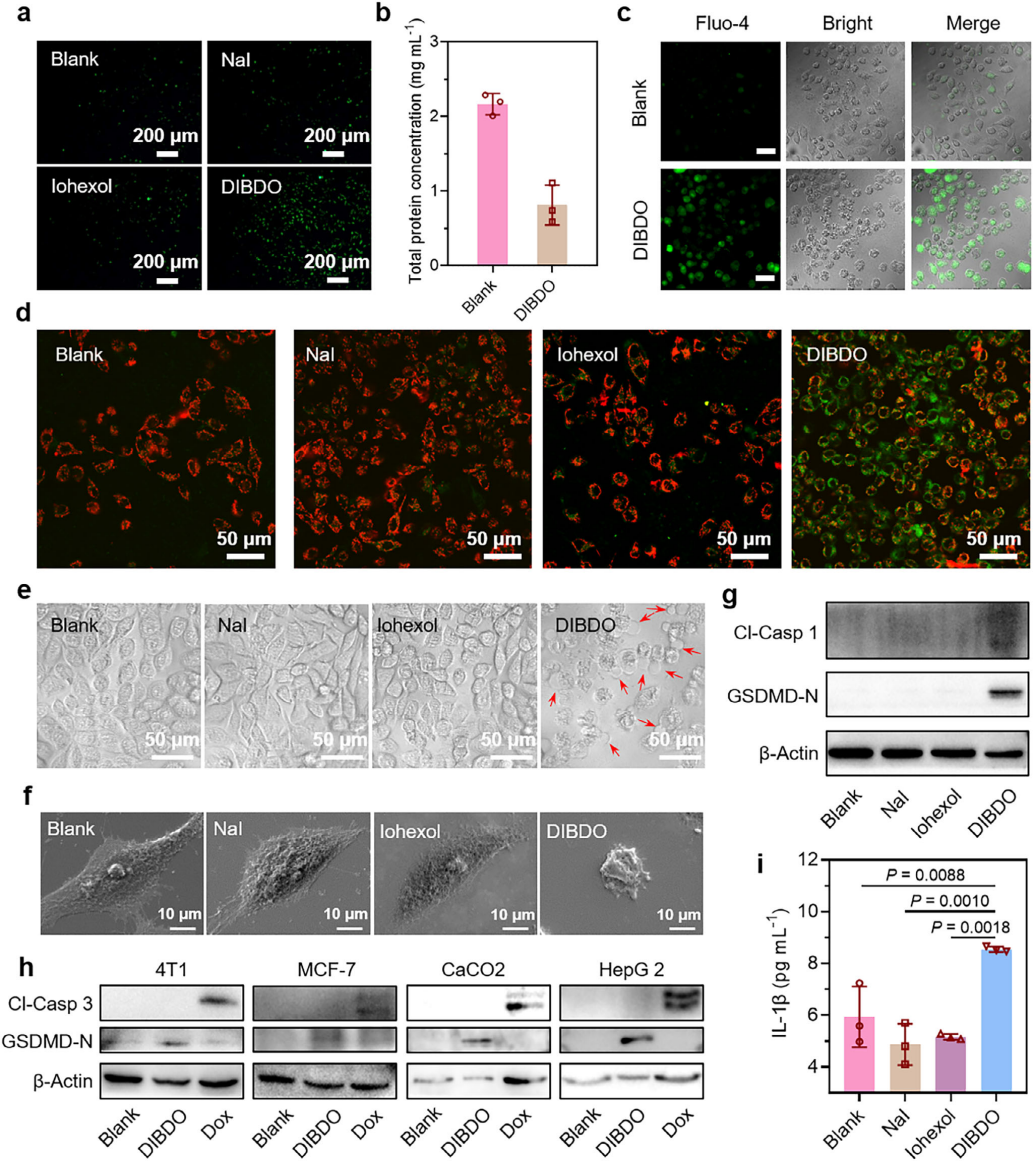
Characterization of DIBDO‐induced tumor cell death pathway. (a) Fluorescence images of HeLa cells treated with NaI (400 µM), iohexol (200 µM), and DIBDO (200 µM), respectively, and stained with DCFH‐DA (n = 3 independent experiments). (b) Total protein concentration of cells treated with or without DIBDO (200 µM) for 4 h (n = 3). (c) Fluorescence images of Fluo‐4 stained HeLa cells treated with or without DIBDO (200 µM) for 4 h. Scale bar: 50 µm. (d) JC‐1 staining of HeLa cells after treated with NaI (400 µM), iohexol (200 µM), and DIBDO (200 µM) for 4 h. (e, f) Optical micrographs (e) and scanning electron microscopy (SEM) images (f) of HeLa cells after treated with NaI (400 µM), iohexol (200 µM), and DIBDO (200 µM) for 12 h. (g) Western blotting analysis of Cl‐Casp‐1 and GSDMD‐N expression in different treatment groups. The treatment condition consistent with those of (e, f). (h) Western blotting analysis of Cl‐casp‐3 and GSDMD‐N in 4T1, MCF‐7, CaCO2, and HepG 2 cells receiving DIBDO (200 µM, 12 h) and Dox (20 µM, 12 h) treatments. (i) IL‐1β release in cell culture supernatants after the cells incubated with NaI (400 µM), iohexol (200 µM), and DIBDO (200 µM), respectively, for 12 h (n = 3, ANOVA). Data = mean ± SD. n = the number of dots as independent replicates in each graph. Statistical analysis was performed using one‐way ANOVA.

Notably, DIBDO‐induced dead cells exhibited pronounced cellular swelling and giant plasma membrane‐derived bubbles (Figure 3e,f), which are typical morphological hallmarks of pyroptosis [7, 54, 55]. In contrast, neither NaI nor iohexol treatment elicited significant morphological changes under identical conditions. Given the established roles of GSDME and GSDMD as pyroptosis executors, we further investigated the molecular pathway of DIBDO‐induced cell death by western blotting. Strikingly, DIBDO‐treated cells showed no detectable GSDME‐N cleavage fragments (Figure S15). Instead, significant GSDMD‐N fragments associated with activated caspase‐1 (Cl‐Casp‐1) were observed after 12 h of DIBDO exposure (Figure 3g and Figure S16). The cleavage of GSDMD as well as the blockade of Casp‐3 activation were also observed across multiple tumor cell lines (Figure 3h and Figure S17) the classical apoptosis inducer doxorubicin (Dox) was employed as a control. These results collectively indicate that DIBDO activates the Casp‐1/GSDMD axis while bypassing Casp‐3 and its downstream apoptotic effectors, establishing GSDMD‐mediated pyroptosis as the dominant cell death mechanism. Concurrently, ELISA revealed 1.75‐fold and 1.65‐fold increases in extracellular IL‐1β release versus NaI and iohexol groups, respectively, indicating membrane permeabilization under DIBDO treatment (Figure 3i).

### 2.4 Potential Mechanism of Specific Cellular Death Pathway

Based on the above experimental findings, a critical question arises how does DIBDO block the activation of Casp‐3 and its downstream pathways. Casp‐3 activation occurs primarily through two distinct mechanisms, intrinsic and extrinsic. The intrinsic pathway involves mitochondrial damage leading to the release of cytochrome c (Cyt C) into cytoplasm [56]. Cyt C assembles with APAF1 in the presence of ATP to cleave Casp‐9 into cleaved‐caspase‐9 (Cl‐Casp‐9) [57, 58, 59], which ultimately cleaves Casp‐3. The extrinsic pathway, which is initiated by stimuli such as necrosis factors, promotes the assembly of Casp‐8, Fas‐associating protein with death domain (FADD), and receptor‐interacting protein kinase 1(RIPK1) into death‐inducing signaling complexes (DISCs) [60, 61], generating cleaved‐caspase‐8 (Cl‐Casp‐8) to subsequently activate Casp‐3. Intriguingly, DIBDO treatment significantly enhanced Cyt C release, yet paradoxically suppressed activation of Casp‐3, Casp‐9, and Casp‐8 (Figure 4a–c, and Figure S18), compared with NaI, iohexol, and a typical apoptosis inducer DOX. This suggests that inhibition of Casp‐9 and Casp‐8 cleavage may be central to DIBDO‐mediated blockade of Casp‐3 activation. Moreover, DIBDO demonstrated superior efficacy in activating GSDMD‐N while suppressing Casp‐3, compared to I_2_, light‐irradiated photosensitizer (Rose Bengal, RB), GSH synthesis inhibitor (buthionine sulfoximine, BSO), and Fe_3_O_4_ (Figure 4d and Figure S19), indicating that ROS generation or GSH inhibition alone is insufficient to inhibit Casp‐3 activation and induce GSDMD‐mediated pyroptosis.

**FIGURE 4:**
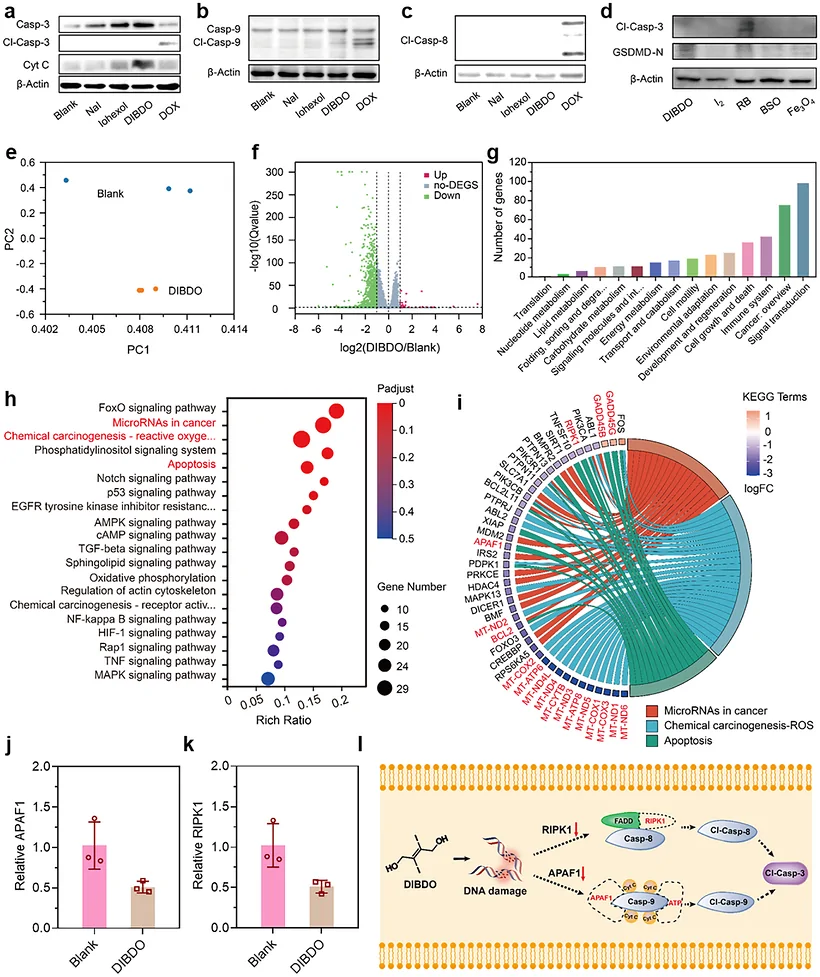
Mechanism of DIBDO‐mediated cell death. (a–c) Western blotting analysis of Cyt C (a), Casp‐9 (b), and Cl‐Casp‐8 (c) in HeLa cells. (d) Western blotting analysis of Cl‐Casp‐3 and GSDMD‐N in cells treated with DIBDO (200 µM), I_2_ (200 µM), RB (32 µM) under 640 nm LED light for 5 min, BSO (100 µM), and Fe_3_O_4_ (100 µg mL^−1^) for 12 h. (e) PCA of blank and DIBDO groups. (f) The volcano plot showing downregulated and upregulated genes in the DIBDO group compared to the blank group. (g) DEGs between the DIBDO and blank groups. (h) KEGG enrichment analysis of DEGs in DIBDO versus blank groups. (i) Enriched chord diagram of KEGG pathways. (j, k) Relative mRNA expression levels of APAF1 (j) and RIPK1 (k) in HeLa cells after treated with DIBDO (200 µM) for 4 h measured by quantitative real‐time polymerase chain reaction (n = 3). (l) Proposed mechanism of Casp‐3 activation downregulation. Data = mean ± SD. n = the number of dots as independent replicates in each graph.

To further dig out the mechanism of DIBDO in cells, genome‐wide RNA sequencing (RNA‐seq) analysis was performed on HeLa cells treated with or without DIBDO. Principal component analysis (PCA) demonstrated distinct gene expression profiles between untreated controls and DIBDO‐exposed groups (Figure 4e). Differential expression analysis identified 29 upregulated genes and 1258 downregulated genes in DIBDO‐treated cells compared to controls (Figure 4f). Reactome pathway annotation of differentially expressed genes (DEGs) revealed significant suppression of signal transduction‐related genes following DIBDO exposure (Figure 4g). Kyoto Encyclopedia of Genes and Genomes (KEGG) pathway enrichment analysis further highlighted that DIBDO predominantly affects genes associated with microRNAs in cancer, chemical carcinogens‐ROS pathway, and apoptosis pathways (Figure 4h). Pathway interrogation (Figure 4i) showed pronounced up‐regulation of DNA damage‐associated genes (GADD45G and GADD45B), aligning with DNA quantification assays. The expression of genes related to mitochondrial respiration (MT‐s) was dramatically downregulated, indicating that ATP synthesis in mitochondria was potently inhibited after DIBDO treatment. Additionally, the expression of genes associated with Casp‐9 and Casp‐8 activation, namely APAF1 and RIPK1, was significantly reduced (Figure 4j,k). The marked reduction in APAF1 and RIPK1 mRNA levels directly impairs Casp 9 and Casp 8 activation, thereby reducing Casp 3 cleavage. Collectively, we derive that internalized DIBDO undergoes GSH‐assisted deiodination and subsequently catalyzes the decomposition of H_2_O_2_, generating substantial ^1^O_2_ and I_2_ that induce oxidative DNA lesions. This subsequently affected ATP synthesis and Casp‐8/‐9 activation, ultimately inhibiting Casp‐3 activation and downstream apoptotic signaling (Figure 4l).

### 2.5 DIBDO Elicits GSDMD‐Mediated Pyroptosis for Enhanced Tumor Immunotherapy In Vivo

Given the high efficacy of DIBDO‐induced pyroptosis, we investigated its potential for in vivo cancer therapy (Figure 5a). At 12 h after the DIBDO treatment, the GSH level in tumor tissues decreased by 3.62‐, 4.66‐, and 3.03 folds compared to control, NaI, and iohexol groups, respectively (Figure 5b and Figure S20). Tumor cells exhibited GSDMD‐mediated pyroptosis at 24 h post treatment (Figure 5c and Figure S21), aligning precisely with in vitro observations. Critically, no significant difference in body weight was observed among all testing groups (Figure S22), suggesting the favorable compatibility of DIBDO. Longitudinal tumor volume monitoring (Figure 5d and Figure S23) revealed that DIBDO treatment significantly suppressed tumor growth, whereas NaI and iohexol exhibited minimal tumor inhibition effects. At the end of monitoring, DIBDO‐treated mice showed the smallest tumor volumes, with one mouse exhibiting complete tumor regression (Figure 5e). Specifically, mean tumor weights of mice in the DIBDO group were reduced to 18%, 32%, and 21% of those in control, NaI, and iohexol groups, respectively (Figure S24). Histological analysis confirmed extensive cell death exclusively in DIBDO‐treated specimens, with no apparent necrosis in control groups (Figure S25). These results establish DIBDO as a potent inducer of sustained pyroptosis‐mediated tumor growth inhibition.

**FIGURE 5:**
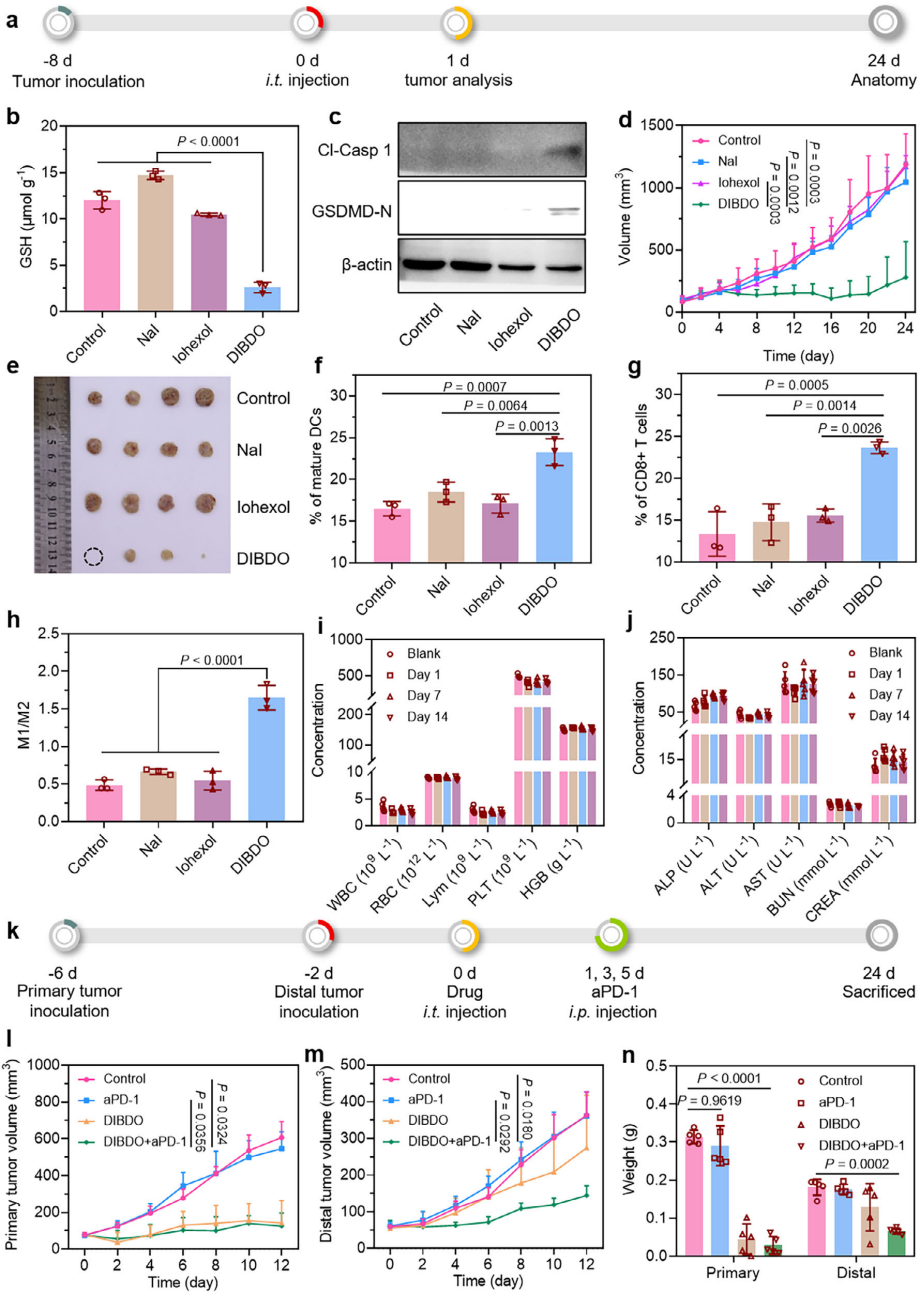
DIBDO‐induced tumor killing and immune activation in vivo. (a) Schematic timeline of in vivo antitumor efficacy experimental time schedule. (b) GSH content in tumor tissue of mice in control, NaI, iohexol, and DIBDO group after treatment (n = 3, ANOVA). Dose: 40 mg/kg. (c) Western blotting analysis of Cl‐Casp‐1 and GSDMD‐N in tumor tissue after varied treatments for 24 h. (d) Time‐dependent tumor volume changing curves for mice in each group (n = 4, ANOVA). (e) Photographs of excised tumors from each mouse in the control, NaI, iohexol, and DIBDO groups after the monitoring period. (f–h) Mature dendritic cells (DCs) (f), CD8^+^ T cells (g), and M1/M2 macrophage ratio (h) in the spleen of mice (n = 3, ANOVA). (i) The concentration of white blood cell (WBC), red blood cell (RBC), blood platelet (PLT), and hemoglobin (HGB) in the blood of mice (n = 5). (j) The concentration of alkaline phosphatase (ALP), alanine aminotransferase (ALT), aspartate aminotransferase (AST), blood urea (BUN), and creatinine (CREA) in the serum of mice. Blank: untreated; Day 1, 7, 14: Mice treated with DIBDO (1 mg per mouse) for respective time before sacrifice (n = 5) (k) Schematic timeline of DIBDO and aPD‐1 synergistic inhibition of distal tumor metastasis. (l, m) Growth curves of primary tumors (l) and distal tumors (m) in varied groups (n = 5, ANOVA). (n) weights of primary tumors and distal tumors across groups at the end of the monitoring (n = 5, ANOVA). DIBDO dose: 40 mg kg^−1^; aPD‐1 dose: 5 mg kg^−1^. Data = mean ± SD. n = the number of dots as independent replicates in each graph. Statistical analysis was performed using one‐way ANOVA.

As GSDMD‐mediated pyroptosis is particularly effective in remodeling immunosuppressive TME and potentiates antitumor immunity [62, 63], we evaluated the immunomodulatory effect of DIBDO‐mediated cell death by investigating the maturation of dendritic cells (DC), infiltration of T cells, and polarization of macrophages in tumor tissue and spleen. After the mice received DIBDO treatment, DC maturation rates increased 41.01% (spleen, Figure 5f and Figure S26a) and 76.57% (tumor, Figure S27a), respectively, and the infiltration of antigen‐specific CD8^+^ T cells also showed a nearly 2‐fold enhancement (Figure 5g, Figures S26b and S27b). Moreover, DIBDO reprogrammed tumor‐associated macrophages from protumoral M2 to antitumoral M1 phenotype, elevating the M1/M2 ratio to 1.67 (Figure 5h and Figure S28), which was 2.44 and 2.76‐fold to that in NaI and iohexol group, respectively. These findings indicate DIBDO promotes cytotoxic T lymphocyte recruitment and converts immunologically “cold” tumors into “hot” immunogenic niches. We also assessed DIBDO's biosafety through hematological and histopathological analyses following intraperitoneal injection (1 mg). No statistically significant differences emerged between DIBDO‐treated and untreated cohorts in complete blood counts (Figure 5i) or hepatic/renal biochemical markers (Figure 5j). H&E staining of major organs (heart, liver, spleen, lung, kidney; Figure S29) revealed no pathological abnormalities or inflammatory lesions in DIBDO‐exposed mice. This comprehensive analysis confirms excellent biocompatibility at the administered dosage, with no adverse effects on hematopoiesis, organ function, or systemic homeostasis.

Building upon the robust immune response induced by DIBDO, we further examined whether combining DIBDO with anti‐PD‐1 (aPD‐1) could enhance systemic therapeutic outcomes and establish the treated primary tumor as an “in situ” vaccine to control distal tumors (Figure 5k). In a bilateral 4T1 tumor‐bearing model, neither monotherapy nor combination therapy (DIBDO+aPD‐1) induced significant body weight change (Figure S30), demonstrating a favorable safety profile. Notably, the mice treated with DIBDO or DIBDO+aPD‐1 effectively suppressed the growth of primary tumors, whereas aPD‐1 alone showed limited therapeutic effect due to the profoundly immunosuppressive tumor environment of 4T1 as a classical “cold tumor” (Figure 5l and Figure S31). Analysis of distal tumors revealed that DIBDO alone showed partial tumor inhibitory effects with marked inter‐individual variability (Figure 5m and Figure S32). In contrast, all mice treated with DIBDO+aPD‐1 achieved sustained suppression of distal tumors (Figure 5m and Figure S33). Overall, the DIBDO+aPD‐1 achieved a tumor inhibition rate of 63.7%, 2.2 times greater than that of DIBDO monotherapy. These results indicated that DIBDO treatment transformed the locally pyroptotic tumor cells into an endogenous vaccine, which synergizes with aPD‐1 immune checkpoint blockade to remodel systemic antitumor immunity. This synergy is crucial in activating T cell‐mediated cross‐lesional immune surveillance and effectively controlling metastatic progression.

### 2.6 DIBDO‐Induced Pyroptotic Cells as Tumor Vaccines

The potent systemic antitumor immunity activation by DIBDO‐induced, GSDMD‐mediated pyroptosis inspired us to evaluate these pyroptotic cells as tumor vaccines. As shown in Figure 6, using an established in vivo vaccination model, mice were subcutaneously immunized with PBS (“Blank” group), liquid nitrogen freeze‐thawed cells (“Control” group), apoptotic Dox‐treated cells (“Dox” group), and pyroptotic DIBDO‐treated cells (“DIBDO” group), respectively. On the 7th day post‐immunization, ten mice in each group received subcutaneous injection of live 4T1 tumor cells into the right flank, and another five mice were injected with live 4T1 tumor cells via intravenous tail vein administration for lung metastasis (Figure 6a). Although no significant body weight differences occurred across groups (Figure 6b), pronounced variations in tumor growth kinetics were observed (Figure 6c, Figure S34). Vaccination with DIBDO‐induced pyroptotic cells substantially suppressed primary tumor progression and elevated the 35‐day survival rate from 0% (blank group) to 90% (Figure 6d). Consistent with this, lung metastatic foci were found in all mice except those treated by DIBDO (Figure 6e). Quantitative analysis confirmed a reduction in pulmonary metastatic nodules of the DIBDO‐treated mice by factors of 5.3, 4.3, and 4.1 relative to the mice in the blank, control, and Dox groups, respectively (Figure 6f). Collectively, these data demonstrate that pyroptotic cells generated via DIBDO induction effectively prime antitumor immunity to restrict 4T1 tumor metastasis.

**FIGURE 6:**
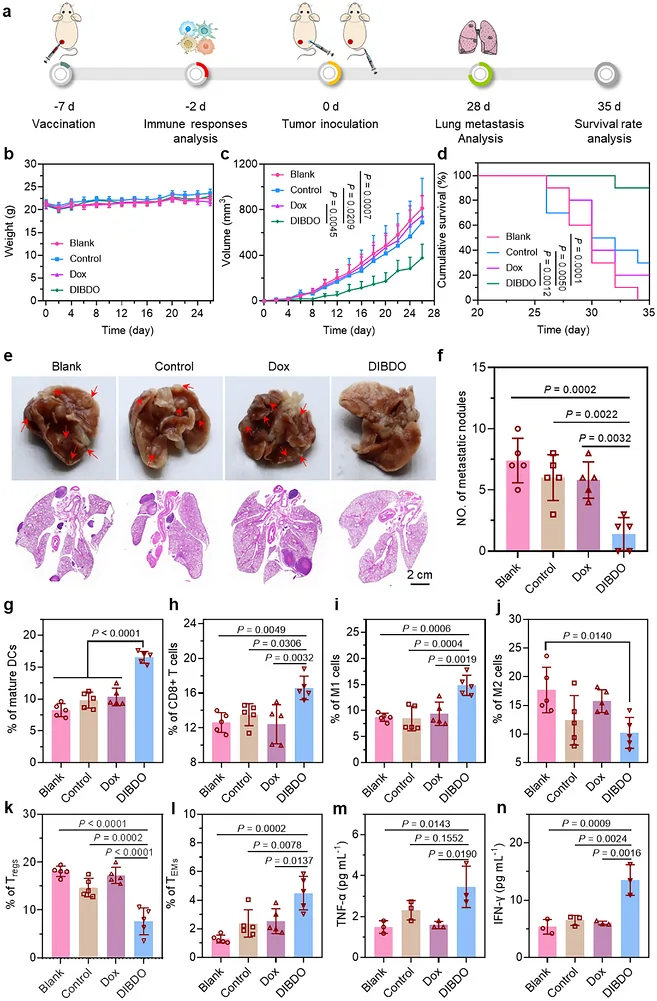
Construction and evaluation of exogenous tumor vaccines based on DIBDO. (a) Scheme of experimental time schedule. (b, c) Time‐dependent body weight changes (b) and tumor volume growth (c) curves for mice in different groups (n = 10, ANOVA). (d) Relative survival curves of mice across treatment groups (n = 10, ANOVA). (e) Representative photograph (upper) and H&E staining (below) of lung tissue from different groups (n = 5). (f) Counting analysis of metastasis nodules in various groups (n = 5, ANOVA). (g–l) Relative quantifications of mDCs (g), CD8^+^ T cells (h), Tumor‐associated M1 macrophages (M1) (i), and Tumor‐associated M2 macrophages (M2) (j), Regulatory T cells (T_regs_) (k), effector memory T cells (*T*_EMs_) (l) in the spleen of mice (n = 5, ANOVA). (m, n) Cytokine levels of TNF‐α (m), and IFN‐γ (n) were isolated from the plasma of the mice in varied treatment groups (n = 3, ANOVA). Data = mean ± SD. n = the number of dots as independent replicates in each graph. Statistical analysis was performed using one‐way ANOVA.

To evaluate the induction of adaptive immunity in mice by DIBDO‐induced pyroptotic cells, we assessed immune remodeling at the cellular level in vaccinated animals. Mice immunized with DIBDO‐induced pyroptotic cells demonstrated DC maturation (Figure 6g), increased infiltration of antigen‐specific CD8^+^ T cells (Figure 6h), and improved macrophage polarization from M2 to M1 (Figure 6i,j and Figures S35 and S36). Concurrently, regulatory T cells (*T*_reg_) populations in the DIBDO‐treated cohort decreased to 7.66% (Figure 6k). In contrast, the population of effector memory T cells (*T*_EM_) reached the highest level, which was 3.5 times of that in the blank group (Figure 6l). This indicated that inoculation of DIBDO‐induced pyroptotic cells established a potent and durable immune memory response. In addition, the TME of the mice vaccinated with DIBDO‐treated cells displayed significantly elevated levels of anti‐tumoral cytokines TNF‐α (Figure 6m) and IFN‐γ (Figure 6n). These results demonstrate that DIBDO‐induced pyroptotic cell death reprograms the TME, depletes *T*_reg_ populations, expands *T*_EM_ cells, and amplifies pro‐inflammatory cytokine signaling, ultimately generating sustained anti‐tumor immune memory.

## 3 Conclusion

In this study, we successfully developed a water‐soluble and electroneutral molecule, DIBDO, that functions as a precise molecular tool to rewire the pyroptotic pathway by bypassing Casp‐3. As a GSH‐activated molecular carrier of iodine, DIBDO releases iodide ions that catalytically generate ^1^O_2_ from H_2_O_2_ in TME, inducing potent oxidative stress. Simultaneously, DIBDO downregulates genes involved in mitochondrial respiration (e.g., MT‐ND1 and MT‐ND6) and Casp‐8/‐9 pathways (e.g., APAF1 and RIPK1), thereby synergistically inhibiting Casp‐3 activation. Under the dual pressure of oxidative stress and suppressed Casp 3 activation, DIBDO shifted the cell death pathway toward GSDMD‐mediated pyroptosis. This dual‐action strategy not only directly induced tumor cell death via effective pyroptosis but also robustly activated immune response, effectively inhibiting tumor growth and metastasis. This work provides a general framework for leveraging specific gasdermin proteins to potentiate cancer immunotherapy, offering a promising and expandable avenue for the development of next‐generation immunogenic cell death inducers. While this study still has several limitations. First, only intratumoral administration was evaluated; systemic delivery and tumor targeting efficiency remain to be explored. Second, all efficacy data were obtained from subcutaneous models; validation in orthotopic or spontaneous metastasis models is needed. Third, long‐term biosafety requires further pharmacokinetic and toxicological investigation. Despite these constraints, the unique mechanism of DIBDO provides a solid foundation for future translational efforts.

## 4 Methods

### 4.1 Materials

2‐Butyne‐1, 4‐diol, glutathione (GSH), NaI, and 3‐(4, 5‐Dimethyl‐2‐Thiazolyl)‐2, 5‐diphenyl tetrazolium bromide (MTT) were sourced from Energy Chemical. 3, 3', 5, 5'‐Tetramethylbenzidine (TMB), dithiobis‐(2‐nitrobenzoic acid) (DTNB) and propidium iodide (PI) were purchased from Shanghai Aladdin Biochemical Technology Co., Ltd. 2’, 7’‐Dichlorodihydrofluorescein diacetate (DCFH‐DA) and 9, 10‐anthracenediyl‐bis(methylene)dimalonic acid (ABDA) were sourced from Sigma–Aldrich. JC‐1 and Hoechst 33342 were acquired from Yuanye Bio‐Technology Co., Ltd (Shanghai). The Annexin V‐FITC/PI Apoptosis Detection Kit and Total Glutathione Assay Kit were purchased from Yeasen Biotechnology (Shanghai) Co., Ltd. and Beyotime Biotechnology, respectively. All solvents were procured from Sinopharm Chemical Reagent Co., Ltd. and used without further purification.

### 4.2 Synthesis of 2, 3‐Diiodobut‐2‐Ene‐1, 4‐Diol (DIBDO)

DIBDO was synthesized as previously reported [41]. Briefly, 3.54 g (13.9 mmol) of molecular iodine was dissolved in tetrahydrofuran (60 mL) and adsorbed onto 10.00 g of dried alumina oxide (98.2 mmol) for 30 min, then 1.00 g (11.6 mmol) 2‐butyne‐1,4‐diol was added and stirred at room temperature for 2.5 h. After filtration and concentration, a crude product was obtained, which was further recrystallized from hot acetone to yield a white DIBDO powder (2.26 g, 57.3%). ^1^H NMR (400 MHz, DMSO‐d6) δ 5.51 (m, 2H), δ 4.20 (d, 4H). ^13^C NMR (400 MHz, DMSO‐d6) δ 105.1, 73.9.

### 4.3 GSH Consumption Measurement

The consumption of GSH by DIBDO was quantified by Ellman's assay. Typically, 20 µL of GSH (reacted with DIBDO for 5 days or unreacted) was mixed with 3 µL of 100 mM DTNB and diluted to 3.0 mL with acetate buffer (0.2 M, pH 6.0). The absorption spectrum of each mixture was recorded by a UV–vis spectrophotometry (TU 1810DSPC, Puxi General Instrumental Co., China).

### 4.4 H_2_O_2_ Consumption Measurement

The consumption of H_2_O_2_ was monitored using TiOSO_4_ as an indicator. Specifically, DIBDO or the reaction product of DIBDO and GSH was combined with 1 mM H_2_O_2_ in 2 mL centrifuge tubes, respectively. All tubes were incubated at 37°C with continuous shaking (150 rpm). At each designated time point, 50 µL aliquot was collected and mixed with 200 µL TiOSO_4_. The absorbance at 405 nm was measured using a Varioskan LUX microplate reader (Thermo Fisher Scientific).

### 4.5 Catalytic Kinetic Measurements

The catalytic activity was assessed using reaction products of DIBDO (2 mM) with GSH at varying concentrations (0.04, 0.1, 0.5, 1, and 10 mM) or reaction durations (0, 6, 12, 24, and 48 h) as catalysts, with TMB (50 µg mL^−1^) as the substrate. Time‐dependent absorbance changes at 652 nm were monitored in acetate buffer (0.2 M) under different pH conditions (6.0, 6.5, 6.8, and 7.4) and H_2_O_2_ concentrations (0.01, 0.1, 1, 10, 100, and 500 mM) using a Varioskan LUX microplate reader (Thermo Fisher Scientific). The catalytic rate was determined from the slope of the absorbance‐time curve.

### 4.6 Detection of ROS

ROS generation and speciation were monitored using TMB (general ROS probe), DCFH‐DA (general ROS probe), ABDA (^1^O_2_ probe), and TA (•OH probe). Specifically, the reaction products of DIBDO with GSH were combined with each probe: TMB (50 µg mL^−1^), DCFH‐DA (20 µM), ABDA (20 µM), or TA (30 µM). After incubation for 3 h at 37°C (150 rpm), the fluorescence spectra were recorded for DCF (Ex: 488 nm, Em: 490 – 600 nm, slit width: 2.5 nm/2.5 nm) and TA (Ex: 315 nm, Em: 320 – 500 nm, slit width: 5.0 nm/5.0 nm). Absorption spectra were acquired for ABDA (300 – 800 nm) and TMB (450 – 850 nm).

### 4.7 Cytotoxicity Assay

Cytotoxicity was evaluated using the MTT assay in cancer cells and normal cells. Taking HeLa cells as an example: cells were seeded in 96‐well plates at a density of 5000 per well and cultured overnight (37°C, 5% CO_2_). The medium was replaced with fresh medium containing DIBDO, DBDO, and NaI (DBDO + 2 eq NaI), or iohexol at varying concentrations. After 24 h of incubation, the medium was removed, and cells were treated with a medium containing MTT solution (0.5 mg mL^−1^) for 4 h. The supernatant was aspirated, and 150 µL of DMSO was added to each well. Absorbance at 570 nm was measured using a microplate reader (Varioskan LUX, Thermo Fisher Scientific).

### 4.8 Intracellular ROS Imaging

Endogenous ROS generation in HeLa cells was monitored using the DCFH‐DA probe. Briefly, HeLa cells were seeded in 96‐well plates at a density of 5000 per well and cultured overnight (37°C, 5% CO_2_). After treatment with PBS, NaI, iohexol, or DIBDO for 4 h, cells were incubated with 10 µM DCFH‐DA (diluted in serum‐free medium) at 37°C for 30 min. The fluorescence images of DCF were then taken using an inverted fluorescent microscope (MF52‐LED, Guangzhou Ming‐Mei Technology Co., Ltd), and the relative fluorescence intensity was quantified using ImageJ software.

### 4.9 JC‐1 Assay

Mitochondrial membrane potential (ΔΨ_m_) was evaluated using the JC‐1 assay. HeLa cells were seeded in 35 mm cell culture dishes and incubated at 37°C for 12 h. Subsequently, the cells were treated with PBS, NaI, iohexol, or DIBDO for 4 h. The medium was then replaced with fresh medium containing JC‐1 (1 µM) and incubated at 37°C for 30 min in the dark. The red fluorescence of J‐aggregates was collected at 580 – 630 nm, while the green fluorescence of monomers was collected at 500 – 550 nm on the Olympus CLSM system (FV3000).

### 4.10 Measurement of IL‐1β Release

Cell culture supernatants from HeLa cells seeded in 12‐well plates were collected to quantify IL‐1β release. Following 12 h of treatment with PBS, NaI, iohexol, or DIBDO, the supernatants were analyzed using a human IL‐1β ELISA kit (BioLegend) according to the manufacturer's protocol.

### 4.11 Western Blotting Assay

The expression levels of apoptosis‐ and pyroptosis‐related proteins were analyzed by Western blotting. Briefly, HeLa cells in 6‐well plates were treated with designated compounds for 12 h, before they were harvested and lysed in ice‐cold RIPA buffer (Beyotime Biotechnology). For Cyt C analysis, the protein was extracted by using Cell Mitochondria Isolation Kit (Beyotime Biotechnology, C3601) following the instructions provided. Protein concentrations were determined using a BCA assay kit. Subsequently, equal amounts of protein were mixed with 5× loading buffer, denatured at 95°C for 5 min, and separated by 12% SDS‐PAGE. Proteins were transferred to a poly(vinylidene fluoride) (PVDF) membrane. Membranes were blocked with 5% BSA in TBST for 1 h at room temperature, then incubated with primary antibodies against Casp‐3 (1:1000, Cell Signaling Technology, 142207T), Cl‐Casp 3 (1:1000, Cell Signaling Technology, 9664T), Cyt C (1:1000, Cell Signaling Technology, 11940T), Casp‐9 (1:1000, Cell Signaling Technology, 9502T), Cl‐Casp‐8 (1:1000, Cell Signaling Technology, 9496T), Casp‐1 (1:1000, Affinity, AF5418), Cl‐Casp‐1 (1:1000, Cell Signaling Technology, 4199T; 1:1000, Affinity, AF4005), GSDMD (1:1000, Affinity, AF4012), GSDME‐N (1:1000, Affinity, AF4016), and β‐actin (1:2000, Solarbio, K023171RR) at 4°C overnight. After three 5‐min TBST washes, membranes were incubated with HRP‐conjugated secondary antibodies (1:5000, Solarbio, SE134) for 1 h at room temperature.

### 4.12 Real‐Time Quantitative PCR (RT‐qPCR) Analysis

Total RNA was isolated using RNA isolater Total RNA Extraction Reagent (Vazyme) in accordance with the manufacturer's protocol. Reverse transcription was performed on 1 µg of mRNA per sample using Hifair III 1st Strand cDNA Synthesis SuperMix (Yeasen) to generate cDNA. Quantitative PCR was subsequently carried out with Hieff UNICON Universal Blue qPCR SYBR Green Master Mix (Yeasen). Primers were obtained from Huayu Gene Biotechnology Co., Ltd. (Wuhan, China), and their sequences are listed in Table 1.

**TABLE 1:** The sequences of the primers.

*APAF1‐forward primer*	*5′‐GGAACAGTGAAGGTATGGAA‐3′*
*APAF1‐reverse primer*	*5′‐CTTGGTAGCATCGTGAGAA‐3′*
*RIPK1‐forward primer*	*5′‐CCACAGAGAAGTCGGATG‐3′*
*RIPK1‐reverse primer*	*5′‐TTATCAACTGCTGCTCACA‐3′*
*GAPDH‐forward primer*	*5′‐GAGTCAACGGATTTGGTCGT‐3′*
*GAPDH‐reverse primer*	*5′‐TTGATTTTGGAGGGATCTCG‐3′*

### 4.13 In Vivo Antitumor Activity

The experiments were approved by the Animal Care and Ethics Committee of Huazhong University of Science and Technology ([2024] IACUC Number: 4785). Female BALB/c mice (8 weeks old) received subcutaneous injections of 4T1 cells (1 × 10^6^ cells in 100 µL PBS) into the right flank. When tumors reached ∼50 mm^3^, mice were randomly divided into four groups and intratumorally injected with NaI, iohexol, and DIBDO. The mixed soybean phosphatidylcholine (SPC) and glycerol dioleate (GDO) were employed to prepare the formulation of therapeutic agents. Tumor size and body weight were recorded every 2 days. The tumor volume was calculated via the formula: volume = 0.5 × length × width^2^.

On day 7 post‐treatment, mice were euthanized. Tumors and spleens were dissociated into single‐cell suspensions. Cells were stained with anti‐CD45‐APC/Cy7, anti‐CD11c‐APC, anti‐CD80‐PE/Cy5, anti‐CD86‐PE, anti‐F4/80‐FITC, anti‐CD11b‐PE/Cy7, anti‐CD206‐Brilliant Violet 605, anti‐CD3‐FITC, anti‐CD4‐Brilliant Violet 421^TM^, and anti‐CD8a‐PE/Cy7. All antibodies were purchased from BioLegend.

To assess the antitumor effect against subcutaneous xenograft distal carcinoma, 100 µL 4T1 cells (1 × 10^6^ cells) were inoculated subcutaneously into the right back of female BALB/c mice. Four days later, 1 × 10^6^ 4T1 cells in 100 µL were injected into the left flank of mice. When primary tumors reached 50 mm^3^, mice were divided into 4 groups: Control (intratumorally injection of SPC/GDO); DIBDO (intratumorally injection of DIBDO@SPC/GDO); aPD‐1 (intraperitoneal injection of aPD‐1); and DIBDO + aPD‐1 (intratumorally injection of DIBDO@SPC/GDO and intraperitoneal injection of aPD‐1). The weight and tumor volume on both sides were monitored every 2 days.

### 4.14 In Vivo Vaccination Experiment

The animal experiments were approved by the Animal Care and Ethics Committee of Huazhong University of Science and Technology ([2024] IACUC Number: 4785). BALB/c male mice (6–8 weeks old) were randomly divided into four groups (20 mice per group): blank group, control group, Dox group, and DIBDO group. In the Dox, or DIBDO groups, the injected cells were pretreated with Dox, or DIBDO, washed, and resuspended in PBS, respectively. In the control group, the cells underwent repeated freeze‐thaw cycles, centrifugation to collect cell corpses, and redispersion of the corpses in PBS. In the blank group, the mice were injected with PBS. The cells or PBS were injected subcutaneously into the left flank of the mice.

Five days later, five mice from each group were sacrificed. Splenocytes were isolated and stained with anti‐CD11c‐APC, anti‐CD80‐PE/Cy5, anti‐CD86‐PE, anti‐F4/80‐FITC, anti‐CD11b‐PE/Cy7, anti‐CD206‐Alexa Fluor 700, anti‐CD3‐FITC, anti‐CD4‐Brilliant Violet 421^TM^, anti‐CD25‐APC, anti‐Foxp3‐Alexa Fluor 700, anti‐CD8a‐PE/Cy7, anti‐CD44‐PE/Cy5, and anti‐CD62L‐PE, and then analyzed by flow cytometry.

On day 7, the remaining mice (n = 10 per group) received a subcutaneous rechallenge with live 4T1 cells (5 × 10^5^ cells) in the right flank. The body weight and tumor volume of the mice were monitored every 2 days. The survival curve of the mice was recorded.

Meanwhile, five mice per group were intravenously injected with 4T1 cells (1 × 10^5^ cells). Three weeks later, the lungs were harvested, fixed in 4% paraformaldehyde (PFA). Tumor metastasizes were counted under a microscope. Lung sections were stained with H&E for histopathological validation.

### 4.15 Statistical Analysis

All experiments were performed in triplicate unless otherwise specified. Unless otherwise specified, the data have not been preprocessed and were presented as means ± SD. Statistical analyses were performed using GraphPad Prism 8.0 software. Multiple‐group comparisons were conducted via one‐way analysis of variance (ANOVA), while comparisons between two groups were assessed using the t‐test. A *p*‐value < 0.05 was considered statistically significant.

## Author Contributions

**Dan Zhao** designed and performed the experiments. **Lingling Zhang**, **Yue Wen**, and **Lianghui Cheng** assisted with the in vivo mouse experiments. **Sidan Tian**, **Fanling Meng**, and **Liang Luo** designed and supervised the study. **Fanling Meng**, and **Liang Luo** conceived the project and secured funding. **Dan Zhao**, **Sidan Tian**, and **Liang Luo** wrote the manuscript. All authors participated in the discussion of the results and provided feedback on the manuscript.

## Funding

This work was financially supported by the National Natural Science Foundation of China (52325304, 21877042, 22077038), the Fundamental Research Funds for the Central Universities, and “Double First‐class” Funds for Central Universities in Hubei (5001170159).

## Ethics Statement

All animal experiments were approved by the Animal Care and Ethics Committee of Huazhong University of Science and Technology ([2024] IACUC Number: 4785), and conducted in accordance with the National Institutes of Health Guide for the Care and Use of Laboratory Animals.

## Conflicts of Interest

The authors declare no conflicts of interest.

## Supporting information




**Supporting File 1**: advs76655‐sup‐0001‐SuppMat.docx.

**Supporting File 2**: advs76655‐sup‐0002‐DataFile.xlsx.

## Data Availability

The data that support the findings of this study are available from the corresponding author upon reasonable request.
