# Supplementary material for: Rewiring Pyroptosis to Potentiate Cancer Immunotherapy via a Gasdermin D Agonist Bypassing Caspase‐3
Source: Adv Sci (Weinh). 2026 Jul 23;13(57):e76655. doi: 10.1002/advs.76655 (PMC13393277; doi:10.1002/advs.76655)
Supplement: Supplementary file 1 [file ADVS-13-e76655-s001.docx]

Supporting Information

Rewiring Pyroptosis to Potentiate Cancer Immunotherapy *via* a Gasdermin D Agonist Bypassing Caspase-3

Dan Zhao, Lingling Zhang, Yue Wen, Lianghui Cheng, Sidan Tian*, Fanling Meng*, Liang Luo*

**Figure S1.** UV-vis absorption spectra of 5,5’-dithiobis-(2-nitrobenzoic acid) (DTNB) in the present of GSH (4 mM) with or without DIBDO (2 mM).

**Figure S2.** Photographs and UV-vis absorption spectra of starch in the present of I_2_ with different concentration.

**Figure S3.** UV-vis absorption spectra of the starch solution in Fig. 1c.

**Figure S4.** Images of AgNO_3_ solution in the present of NaI (4 mM), DIBDO (2 mM), and DIBDO + GSH. 2 mM of DIBDO was co-incubated with 4 mM of GSH for 5 days in PB (0.1 mM, pH = 6.0).

**Figure S5.** Fluorescence spectra of 2’,7’-dichlorodihydrofluorescein (DCF) after different treatments. DCF: 20 μM; H_2_O_2_: 500 mM; DIBDO+GSH was prepared by incubating DIBDO (2 mM) with GSH (10 mM) for 5 days. The mixture aliquot was diluted by acetic acid/sodium acetate buffer (pH 3.6, 0.2 M) for 20 times for ROS measurement.

**Figure S6.** Time-dependent remaining level of H_2_O_2_ in the presence of DIBDO/GSH mixture (molar ratio 1:5) at different DIBDO concentrations (n = 3). Data = mean ± SD. n = the number of dots as independent replicates in each graph.

**Figure S7.** Time-dependent absorbance changes at 652 nm for the reaction mixture of DIBDO (2 mM) and GSH (10 mM) for 5 days, which was diluted by different media for 20 times, in the presence of TMB (50 μg mL^-1^) and H_2_O_2_ (500 mM).

**Figure S****8.** a) UV-vis absorption spectra of 9,10-anthracenediyl-bis(methylene)dimalonic acid (ABDA, 20 μM) incubated with H_2_O_2_, NaI, and NaI + H_2_O_2_. NaI concentration: 200 μM; H_2_O_2_ concentration: 500 mM. b) UV-vis absorption spectra of ABDA (20 μM) incubated with H_2_O_2_, DIBDO + GSH, and DIBDO + GSH + H_2_O_2_. DIBDO + GSH was prepared by co-incubated DIBDO (2 mM) with GSH (10 mM) for 5 days; During testing, the DIBDO concentration was 100 μM. c) Fluorescence spectra of terephthalic acid (TA, 50 μM) in the present of H_2_O_2_, DIBDO + GSH, FeSO_4_, DIBDO + GSH + H_2_O_2_, and FeSO_4_ + H_2_O_2_. FeSO_4_ concentration: 100 μM.

**Figure S9.** a) Time-dependent absorbance changes at 652 nm for the mixed solution of TMB (50 μg mL^-1^) and H_2_O_2_ (500 mM), in the presence of DIBDO (50 μM) or DBDO (50 μM). Absorption spectra (b) and the time-dependent absorbance change at 652 nm (c) for the mixed solution of TMB (50 μg mL^-1^) and H_2_O_2_ (500 mM), in the presence of NaI with different concentration. d) Slope of the time-dependent absorbance change at different NaI concentrations.

**Figure S10.** Catalytic activity of iohexol (2 mM) treated with or without GSH (10 mM) for different time (n = 3). Data = mean ± SD. n = the number of dots as independent replicates in each graph.

**Figure S11.** Time-dependent absorbance (a) and increased absorbance (b) at 652 nm for the mixed solution of TMB (50 μg mL^-1^), H_2_O_2_ (500 mM), and cell lysate solution when the cells were pretreated with H_2_O, NaI (2 mM), iohexol (1 mM), and DIBDO (1 mM) (n = 3). Data = mean ± SD. n = the number of dots as independent replicates in each graph.

**Figure S12.** Corresponding quantitative analysis of HeLa cells stained with 2’,7’-dichlorodihydrofluorescein diacetate (DCFH-DA) in Fig. 3a (n = 3). Data = mean ± SD. n = the number of dots as independent replicates in each graph. Statistical analysis was performed using one-way ANOVA.

**Figure S13.** Genome DNA concentration of cells treated with or without DIBDO (200 μM) for 4 h (n = 3). Data = mean ± SD. n = the number of dots as independent replicates in each graph.

**Figure S14.** Corresponding quantitative analysis of fluorescence intensity when HeLa cells treated with or without DIBDO (200 μM) for 4 h and then stained with Fluor-4 AM (n = 3). Data = mean ± SD. n = the number of dots as independent replicates in each graph.

**Figure S15.** Western blot results of GSDME-N in HeLa cells after treated with NaI (400 μM), iohexol (200 μM), DIBDO (200 μM), and Dox (20 μM) for 12 h.

**Figure S****16.** Uncropped Western blot results of Cl-Casp-1, GSDMD-N, and β-Actin in cells after treated with NaI (400 μM), iohexol (200 μM), and DIBDO (200 μM) for 12 h.

**Figure S****17.** Uncropped western blotting analysis of Cl-Casp-3 and GSDMD-N in 4T1, MCF-7, CaCO2, and HepG 2 cells receiving various treatments.

**Figure S****18.** Uncropped Western blot results of Cyt C, Cl-Casp-9, and Cl-Casp-8 in cells after various treatments.

**Figure S****19.** Uncropped Western blot results of Cl-Casp-3, GSDMD-N, and β-Actin in cells after various treatments.

**Figure S20.** Uv-vis absorption spectra of DTNB incubated with tumor lysates after various treatments for 12 h.

**Figure S21.** Uncropped Western blot results of Cl-Casp-1, GSDMD-N, and β-Actin in tumors after various treatments.

**Figure S22.** Time-dependent body weight changing curves for mice in different groups (n = 4 independent biological samples). Data = mean ± SD. n = the number of dots as independent replicates in each graph.

**Figure S23.** Time-dependent tumor volume changing curves for each mouse in group of Control (a), NaI (b), iohexol (c), and DIBDO (d).

**Figure S24.** The weights of dissected tumors from the mice in different groups (n = 4 independent biological samples). Data = mean ± SD. n = the number of dots as independent replicates in each graph.

**Figure S25.** Histological analysis of tumor tissues in each group by hematoxylin and eosin (H&E) staining and terminal deoxynucleotidyl transferase-mediated dUTP nick-end labeling (TUNEL) assay.

**Figure S****26.** Representative flow cytometry analysis of mature DCs (CD11c^+^CD80^+^CD86^+^) (**a**) and CD8^+^ T cells (CD3^+^CD8^+^) (**b**) in spleen of mice (n = 3 independent biological samples).

**Figure S27.** Representative flow cytometry analysis and corresponding quantification of mature DCs (CD11c^+^CD80^+^CD86^+^) (**a**) and CD8^+^ T cells (CD3^+^CD8^+^) (**b**) in tumor of mice. (n = 3 independent biological samples). Data = mean ± SD. n = the number of dots as independent replicates in each graph.

**Figure S28.** Corresponding quantification of M1 cells (CD11b^+^CD86^+^) (**a**), and M2 cells (CD11b^+^CD206^+^) (**b**) in spleen of mice (n = 3 independent biological samples, ANOVA). Data = mean ± SD. n = the number of dots as independent replicates in each graph.

**Figure S29.** Representative photographs of H&E stained tissue sections of heart, liver, spleen, lung, and kidney. Scale bar: 100 μm.

**Figure S30.** Time-dependent body weight changing curves for mice in different treatment groups (n = 5 independent biological samples). Data = mean ± SD. n = the number of dots as independent replicates in each graph.

**Figure S31.** Volume growth curve of each primary tumor in Control, aPD-1, DIBDO, and DIBDO + aPD-1 groups.

**Figure S32.** Volume growth curve of each distal tumor in Control, aPD-1, DIBDO, and DIBDO + aPD-1 groups.

**Figure S33.** Photos of primary and distal tumors dissected from for mice in different treatment groups.

**Figure S****34.** Time-dependent tumor volume changing curves for each mouse in group of blank (**a**), control (**b**), Dox (**c**), and DIBDO (**d**).

**Figure S35.** Representative flow cytometry analysis and corresponding quantification of mature DCs (CD11c^+^CD80^+^CD86^+^) (**a**), M1 cells (CD11b^+^CD86^+^) (**b**), M2 cells (CD11b^+^CD206^+^) (**c**), TEM (CD11b^+^CD8^+^CD44^+^CD62L^-^) (**d**), and Treg (CD4^+^CD25^+^Foxp3^+^) (**e**) in spleen of mice from 5 independent biological samples.

**Figure S****36.** M1/M2 macrophage ratio in spleen of mice (n = 5). Data = mean ± SD. n = the number of dots as independent replicates in each graph. Statistical analysis was performed using one-way ANOVA.
